# Association Between ABO or Rh Blood Groups and Chikungunya Virus Infection: A Systematic Review and Meta-Analysis

**DOI:** 10.3390/medicina61081316

**Published:** 2025-07-22

**Authors:** Yanisa Rattanapan, Wanatsanan Chulrik, Karunaithas Rasaratnam, Thitinat Duangchan

**Affiliations:** 1 Medical Technology, School of Allied Health Sciences, Walailak University, Nakhon Si Thammarat 80160, Thailand; yanisa.rt@wu.ac.th (Y.R.); wanatsanan.chu@wu.ac.th (W.C.); 2 Hematology and Transfusion Science Research Center, Walailak University, Nakhon Si Thammarat 80160, Thailand; 3 Food Technology and Innovation Research Center of Excellence, Research and Innovation Institute of Excellence, Walailak University, Nakhon Si Thammarat 80160, Thailand; 4 Department of Medical Laboratory Sciences, Faculty of Allied Health Sciences, University of Jaffna, Jaffna 40000, Sri Lanka; karunaithas@univ.jfn.ac.lk

**Keywords:** ABO, blood groups, Chikungunya virus, Rhesus, susceptibility

## Abstract

*Background and Objectives*: The relationship between ABO or Rh blood groups and susceptibility to Chikungunya virus (CHIKV) infection remains unclear. This systematic review and meta-analysis aimed to synthesize available evidence on this association. *Materials and Methods*: Studies reporting ABO and/or Rh blood groups and CHIKV infection were searched through PubMed, Scopus, EMBASE, MEDLINE, Ovid, ProQuest, and Google Scholar up to 8 July 2025. A random-effects meta-analysis was conducted to calculate pooled odds ratios (Ors) with 95% CIs. Heterogeneity was assessed using *I*^2^ statistics. Subgroup analyses were performed based on study design and study quality. Sensitivity analysis was conducted using a leave-one-out method. Publication bias was evaluated via funnel plots and Egger’s test. *Results*: Seven studies, including 24,828 participants, were included. No significant associations were observed between blood groups A, B, AB, or Rh(D) and CHIKV infection. However, blood group O was significantly associated with an increased risk of CHIKV infection (OR: 1.52, 95% CI: 1.01–2.29, *p* = 0.043, *I*^2^ = 95.38%) compared to non-O blood groups. Subgroup analyses showed stable results. Nevertheless, the sensitivity analysis indicated that certain studies had a greater influence on the overall results. In addition, significant publication bias was also detected. *Conclusions*: Current evidence indicates that blood group O is significantly associated with an increased susceptibility to CHIKV infection. In contrast, no consistent associations were observed for other ABO or Rh blood groups. Due to substantial heterogeneity and methodological limitations, these findings should be interpreted with caution. Further well-designed, large-scale studies with standardized diagnostics are needed to clarify these associations and underlying mechanisms.

## 1. Introduction

Chikungunya virus (CHIKV) is an arthropod-borne alphavirus in the family Togaviridae, primarily transmitted by *Aedes aegypti* and *Aedes albopictus* mosquitoes [1] CHIKV infection typically presents as an acute febrile illness accompanied by high fever, rash, and incapacitating polyarthralgia. Although it is rarely fatal, it can lead to prolonged joint pain and chronic fatigue, substantially impairing quality of life [2]. Chikungunya management focuses on symptom relief, as there is no specific antiviral treatment available. This involves rest, fluid intake, and over-the-counter pain relievers to control fever and pain. In some cases, nonsteroidal anti-inflammatory drugs (NSAIDs) and corticosteroids may be prescribed to alleviate persistent joint pain, often with physical therapy [3].

Over the past decades, CHIKV has caused numerous outbreaks across Africa, Asia, and the Americas [4]. As of December 2024, a total of 119 countries and territories have documented evidence of CHIKV transmission [5]. In Africa, a marked increase in CHIKV transmission was first observed during an outbreak in Kenya in 2004. The largest outbreak occurred on La Reunion, a French department in the Indian Ocean, from 2005 to 2006, with an estimated 244,000 infections [6]. In 2020, Zambia reported a CHIKV prevalence rate of 36.9% (95% CI: 30.5–43.4) [7]. In the Americas, CHIKV transmission was first detected in December 2013 on the Caribbean island of Saint Martin and quickly spread to other islands, including Martinique and Guadeloupe in the French West Indies [8]. By 2014, the epidemic had affected a large portion of the population, with seroprevalence rates reaching up to 60% in some areas [9]. In Nicaragua, the first imported case was detected in July 2014. The overall clinical attack rate of laboratory-confirmed CHIKV infection in children was 2.9% (95% CI: 2.3–3.4) [10]. Chikungunya is endemic in Thailand, with sporadic outbreaks reported. Between 2018 and 2020, Thailand experienced a large outbreak of more than 27,000 Chikungunya cases across 60 provinces [11]. In India, the annual cumulative number of confirmed cases ranged from 43,424 to 81,914 between 2018 and 2020, with over 115,000 suspected cases reported each year from 2021 to 2023 [12]. CHIKV first emerged in Bangladesh in 2008, followed by significant outbreaks in subsequent years, with the largest occurring in 2017, when over 2 million suspected cases were recorded [13].

The ABO and Rh blood group systems are among the most thoroughly studied genetic polymorphisms in humans. While primarily recognized for their critical role in transfusion medicine, increasing evidence has linked these blood group antigens to susceptibility to various infectious diseases [14]. Associations have been reported between ABO/Rh blood groups and infections such as *Plasmodium falciparum* malaria, norovirus, hepatitis B virus, and SARS-CoV-2 [15,16,17]. For example, the odds of developing severe malarial anemia were 16 times higher in children under 12 years of age and 17.8 times higher in patients aged ≥12 years with blood group A [18]. Individuals with blood group O were found to have increased odds of norovirus infection (OR = 1.28, 95% CI: 1.03–1.59, *p* = 0.03) [19]. Additionally, individuals with B, Rh(D)-negative status were associated with HBV infection (OR = 20.174, 95% CI: 0.800–536.26, *p* = 0.059) [20]. Conversely, a lower risk for severe COVID-19 illness or death has been observed in individuals with blood group O compared to all other blood groups [21].

In the context of CHIKV, research exploring the influence of ABO and Rh blood groups has only recently emerged. A study in Andhra Pradesh, India, found that individuals with Rh-positive blood, particularly those with blood groups AB and A, had a significantly increased risk of CHIKV infection. The reported odds ratios (ORs) were 3.03 for group AB and 2.83 for group A, compared to Rh-negative individuals [22]. This observation suggests that erythrocyte surface antigens may play a role in virus–host interactions or modulating host immune responses. In contrast, a recent study in Bangladesh reported the highest infection rate among individuals with blood group O (49.3%) and the lowest among those with blood group AB (9%). After adjusting for blood group distribution in the population, individuals with blood groups B, O, and AB showed higher susceptibility than those with group A [23]. These findings highlight potential population-level variability and the need for region-specific investigations. Conversely, some studies have reported no significant association between ABO or Rh blood groups and CHIKV infection risk. A study conducted in Thailand found no consistent correlation between ABO blood group phenotypes and the incidence of Chikungunya fever, underscoring the likely involvement of other genetic or environmental factors [24].

These contrasting findings reflect the complexity of host–pathogen interactions and emphasize the need for large-scale, population-specific studies. A deeper understanding of the role of ABO and Rh blood groups in CHIKV susceptibility may contribute to risk stratification, inform epidemiological modeling, and enhance targeted prevention strategies. The aim of this review was to elucidate the association between ABO or Rh blood groups and Chikungunya virus infection, providing a comprehensive analysis of the existing evidence to determine whether these genetic factors play a significant role in determining susceptibility to CHIKV.

## 2. Materials and Methods

### 2.1. Registered Protocol

The protocol guiding this review was prospectively registered in PROSPERO (ID: CRD42024551401). The systematic review was conducted in accordance with the PRISMA guidelines [25], and the research question was framed using the Population, Exposure, and Outcome (PEO) framework. The PEO framework was employed to determine the relationship between specific risk factors (individuals with ABO or Rh blood groups) and the outcome of CHIKV infection [26].

### 2.2. Search Strategy

Searching was performed on 23 January 2025, using five electronic databases: PubMed, Scopus, EMBASE, MEDLINE, and Ovid. ProQuest and Google Scholar were additionally searched on 8 July 2025. No restrictions were applied regarding language or publication date. The search strategy, including MeSH terms and Boolean operators, is detailed in Table S1.

### 2.3. Selection of Studies

All observational studies that reported the occurrence of CHIKV infection confirmed through serological or symptom-based diagnostic methods were included in this study. Studies also had to provide ABO and/or Rh blood group data for participants with and without CHIKV infection. Studies with missing or unclear data on blood group or CHIKV diagnosis were excluded. In addition, case reports, reviews, and articles lacking relevant data or full-text access were excluded. The selection of studies was performed independently by two authors (YR and WC), and any discrepancies were resolved through discussion with a third author (TD).

### 2.4. Data Extraction

Data were independently extracted by two authors (YR and WC) using a standardized form in Microsoft Excel (Microsoft Corporation, Redmond, WA, USA). The extracted data included the main outcomes: sample size and number of participants in each blood group with and without CHIKV infection. Additionally, data were sought for other variables, including first author, publication year, country, study design, participant characteristics, diagnostic methods for CHIKV infection and blood group analysis. Any discrepancies were resolved through discussion with a third author (TD).

### 2.5. Quality Assessment

The quality of the included studies was assessed using the Strengthening the Reporting of Observational Studies in Epidemiology (STROBE) checklist for observational studies [27]. This checklist evaluates 22 key reporting components across different study designs, including cross-sectional, cohort, and case-control studies. Each checklist item was scored as 1 (present) or 0 (absent). Based on the total score percentile, studies were classified into three categories: high (>75), moderate (50–75), and low quality (<50). The quality assessment was performed independently by two authors (YR and WC), with discrepancies resolved through discussion with a third author (TD).

### 2.6. Data Analysis

A meta-analysis using a random-effects model was performed to compute the combined log odds ratio (OR) comparing the risk of CHIKV infection between individuals with blood groups A vs. non-A, B vs. non-B, AB vs. non-AB, O vs. non-O, as well as between Rh-positive and Rh-negative individuals, with 95% confidence intervals (CIs). The random-effects model was chosen due to anticipated heterogeneity between studies [28]. The degree of heterogeneity was assessed using the inconsistency index (*I*^2^), with thresholds of 25%, 50%, and 75% representing low, moderate, and high heterogeneity, respectively [29]. Subgroup analysis was conducted based on factors, including study design and study quality. Sensitivity analysis was performed using a leave-one-out approach to assess the impact of individual studies on the pooled estimate. Publication bias was evaluated visually using funnel plots and quantitatively assessed by Egger’s regression test. All statistical analyses were performed using Stata 18.0 (StataCorp LLC, College Station, TX, USA), with statistical significance defined as a *p*-value < 0.05.

## 3. Results

### 3.1. Search Results

**Figure 1:**
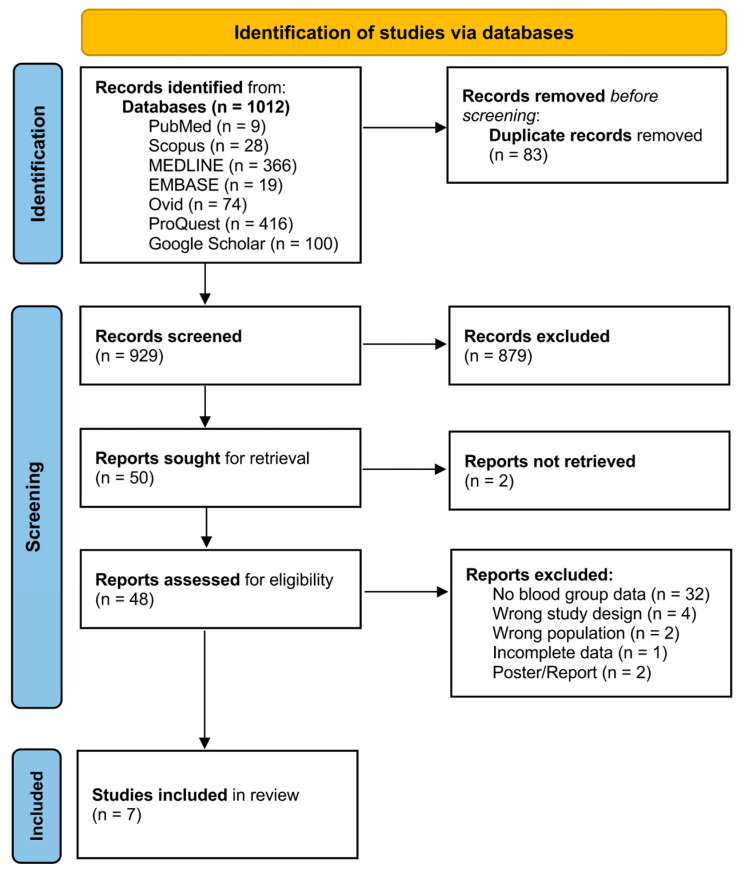
PRISMA flow diagram of study selection.

A total of 1012 records were initially identified through database searches, including PubMed (n = 9), Scopus (n = 28), MEDLINE (n = 366), EMBASE (n = 19), Ovid (n = 74), ProQuest (n = 416), and Google Scholar (n = 100). After removing 83 duplicates, 929 studies remained for screening based on their titles and abstracts. Of these, 879 studies were excluded due to irrelevance to the research question. A total of 50 reports were sought for retrieval, with 2 reports not retrieved. Ultimately, 48 full-text articles were then assessed for eligibility. Of these, 32 reports were excluded due to the absence of blood group data, 4 due to wrong study design, 2 for wrong population, 1 due to incomplete data, and 2 for wrong publication types (poster/report). Finally, a total of 7 articles were included in this review [22,23,24,30,31,32,33]. Flow diagram of the study selection process is shown in Figure 1.

### 3.2. Characteristics of the Included Studies

Among the included studies, four were conducted in Asia, including one from Thailand, two from India, and one from Bangladesh. In addition, one study was conducted in the French West Indies, one in Nicaragua, and one in Zambia. These studies were published between 2009 and 2024. Four of the studies were cross-sectional in design, while the other three were case-control studies. Based on the quality assessment using the STROBE criteria, five studies were considered as high quality, one as moderate quality, and one as low quality (Table S2).

In total, 24,828 participants were included across the seven studies. Of these, 4062 (16%) participants were reported to be positive for CHIKV infection, while 20,766 (84%) were negative. The presence of CHIKV infection was primarily confirmed through serological testing for anti-CHIKV antibodies. Regarding ABO blood group distribution, group O was the most prevalent, comprising 41% (n = 10,201), followed by group B at 26% (n = 6332), group A at 25% (n = 6159), and group AB at 9% (n = 2136), respectively. For the Rh factor, Rh-positive individuals were more prominent, making up 94% (n = 23,160), while Rh-negative individuals accounted for 6% (n = 1401). The ABO and Rh blood groups were primarily determined using the hemagglutination test (Table S3).

### 3.3. Association Between ABO Blood Group and Chikungunya Virus Infection

**Figure 2:**
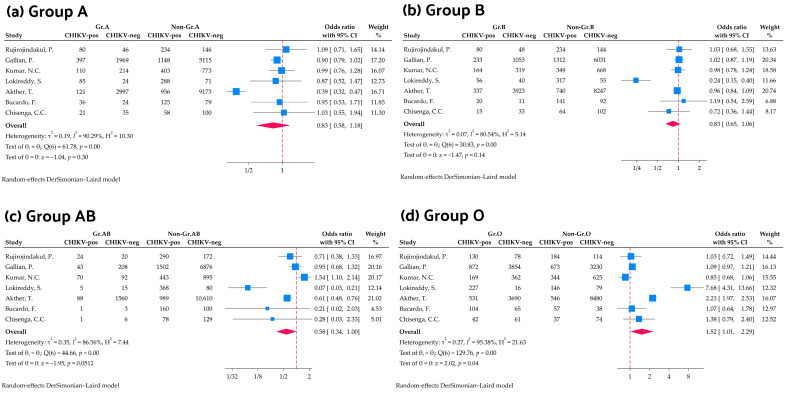
Forest plots illustrate the odds of CHIKV infection among individuals with blood groups (**a**) A, (**b**) B, (**c**) AB, and (**d**) O using a random-effects model [22,23,24,30,31,32,33].

A total of 24,828 participants from seven studies were included in the meta-analysis examining the association between ABO blood group and CHIKV infection. The results showed that individuals with blood group A had no greater odds of CHIKV infection compared to individuals who were non-group A (OR: 0.83, 95% CI: 0.58–1.18, *p* = 0.30, *I*^2^ = 90.29%). Similarly, for blood group B, no significant association with CHIKV infection was found when compared to non-B blood groups (OR: 0.83, 95% CI: 0.65–1.06, *p* = 0.14, *I*^2^ = 80.54%). Interestingly, blood group AB showed a slight resistance to CHIKV infection, although no significant difference was observed (OR: 0.58, 95% CI: 0.34–1.00, *p* = 0.051, *I*^2^ = 86.56%). In contrast, blood group O was associated with an increased risk of Chikungunya infection compared to non-O blood groups (OR: 1.52, 95% CI: 1.01–2.29, *p* = 0.043, *I*^2^ = 95.38%). The high *I*^2^ values for each ABO blood group indicate a substantial degree of heterogeneity among the studies (Figure 2).

### 3.4. Association Between Rh Blood Group and Chikungunya Virus Infection

**Figure 3:**
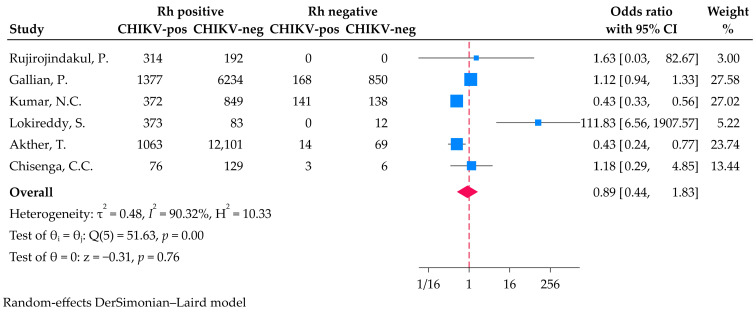
Forest plot illustrates the odds of CHIKV infection among individuals with Rh-positive and Rh-negative blood groups using a random-effects model [22,23,24,30,31,33].

A total of 24,561 participants from six studies were included in the investigation of the association between Rh blood group and CHIKV infection. The meta-analysis found no difference in the odds of having CHIKV infection between the Rh-positive and Rh-negative individuals. The pooled OR was 0.89 (95% CI: 0.44–1.83, *p* = 0.76). The *I*^2^ value of 90.32% suggests a high degree of heterogeneity among the studies (Figure 3).

### 3.5. Subgroup Analysis

Due to the high degree of heterogeneity observed among the included studies, a subgroup analysis was conducted to explore the potential sources of heterogeneity in the association between ABO or Rh blood groups and CHIKV infection, based on study design (case-control vs. cross-sectional) and study quality (low vs. high). The results for group differences did not show significant differences between subgroups (*p* > 0.05) This suggests that study design and study quality do not modify the risk of CHIKV infection in individuals with blood groups A, B, AB, O, and Rh(D) (Figures S1–S3).

### 3.6. Sensitivity Analysis

**Figure 4:**
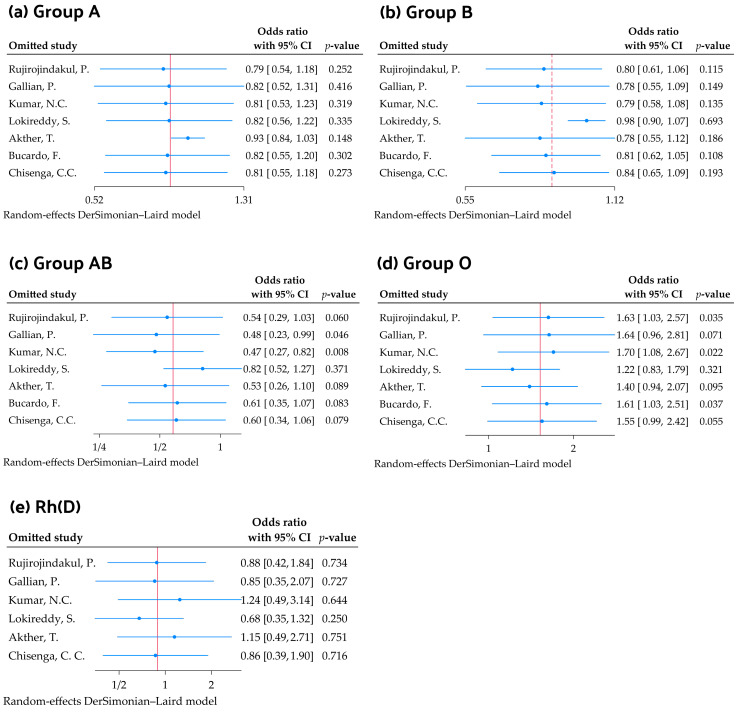
Sensitivity analysis using the leave-one-out method demonstrates the effects of each individual study on the meta-analysis results for blood groups (**a**) A, (**b**) B, (**c**) AB, (**d**) O, and (**e**) Rh(D) [22,23,24,30,31,32,33].

The sensitivity analysis was performed using the leave-one-out method to assess the stability of the pooled ORs for ABO and Rh blood groups by omitting one study at a time. The analysis showed that the pooled ORs for blood groups A and B remained stable and non-significant across most studies. However, for blood group AB, omitting Gallian et al. [30] and Kumar et al. [22] caused significant changes, with the pooled OR decreasing to 0.48 (95% CI: 0.23–0.99, *p* = 0.046) and 0.47 (95% CI: 0.27–0.82, *p* = 0.008), respectively. For blood group O, the results exhibited a notable variation depending on the study excluded. For example, when Kumar et al. [22] and Rujirojindakul et al. [24] were omitted, the pooled OR increased to 1.70 (95% CI: 1.08–2.67, *p* = 0.022) and 1.63 (95% CI: 1.03–2.57, *p* = 0.035), respectively. However, when Lokireddy et al. [31] was omitted, the pooled OR decreased to 1.22 (95% CI: 0.83–1.79), with a *p*-value of 0.321. The Rh blood group analysis showed consistency across studies, with no major effect from omitting any individual study (Figure 4).

### 3.7. Publication Bias

Publication bias for ABO and Rh blood groups was determined using funnel plots, and small-study effects were evaluated through Egger’s test. No publication bias was observed for blood groups A, B, and O (*p* = 0.4346, 0.2268, and 0.2730, respectively). However, asymmetric funnel plots were found for blood groups AB and Rh, with significant small-study effects (*p* = 0.0354 and 0.02919, respectively). These results suggest that some studies may be missing from the meta-analysis, contributing to the observed asymmetry (Figure S4).

## 4. Discussion

This systematic review and meta-analysis is the first study to synthesize available evidence on the association between ABO or Rh blood groups and susceptibility to CHIKV infection. Seven observational studies consisting of 24,828 participants were included in this review. The results indicate no statistically significant association between blood groups A, B, AB, or Rh factor and CHIKV infection. Interestingly, blood group O showed a significantly increased risk, but a high degree of heterogeneity was observed among the included studies, which reduces certainty in this finding. Additionally, publication bias for blood groups AB and Rh(D) was identified through visual inspection of funnel plots and Egger’s test results.

Previous studies have identified associations between ABO blood groups and susceptibility to infectious diseases, including malaria, dengue, and COVID-19 [34,35,36]. Possible mechanisms explaining such associations include the role of blood group antigens as viral receptors or co-receptors, modulation of inflammatory responses, as well as their influence on cytokine-mediated immune responses [37,38]. For instance, individuals with blood group O generally exhibit lower levels of von Willebrand factor and interleukin-6 (IL-6), both of which may affect viral pathogenesis or immune-mediated severity of disease [39]. In addition, blood group O has been associated with protection against *Plasmodium falciparum* malaria due to reduced rosetting of infected erythrocytes [15]. For viral infections, blood group A has been linked to increased susceptibility to SARS-CoV-2 and SARS-CoV-1, potentially through interactions with ACE2 and anti-A isoagglutinin that inhibit viral entry [40]. Similarly, studies on viruses such as norovirus and rotavirus have demonstrated that specific blood group antigens influence viral attachment and host susceptibility [41,42]. Moreover, in norovirus infection, host secretor status and blood group antigens determine susceptibility, with non-secretors showing natural resistance [43].

Though direct evidence for CHIKV is limited, it is plausible that the absence of A and B antigens on erythrocytes and epithelial cells in group O individuals may alter viral binding dynamics, immune recognition, or inflammatory response [44,45]. Additionally, our findings showed no association between the Rh(D) antigen and CHIKV infection. This is consistent with the biological understanding that the Rh(D) antigen is strictly confined to red blood cells and may not be involved in the risk of virus–host interactions [46].

Notably, inconsistencies across the included studies could be issues. For example, Kumar et al. [22] reported a higher risk for blood groups AB and A Rh-positive individuals in India, whereas Akther et al. [23] and Lokireddy et al. [31] found blood group O to be most frequently affected in Bangladesh and India, respectively. These inconsistent results could reflect regional differences in allele frequencies, host genetic backgrounds, environmental exposures, or circulating CHIKV genotypes [15]. Although the largest study included in this review [23], involving 13,247 participants, showed a positive association between group O and CHIKV infection, its influence on the pooled estimate was limited by the random-effects model, which balances weights between large and small studies [47]. Additionally, methodological limitations such as self-reported blood groups and symptom-based CHIKV diagnosis in Kumar et al. [22] and Lokireddy et al. [31] raise concerns about data reliability.

The substantial heterogeneity observed in all pooled analyses (*I*^2^ > 80%) highlights sources of variability, including variability in diagnostic methods, differences in population structure, and blood group distribution. Despite conducting subgroup analyses based on study design (case-control vs. cross-sectional) and study quality (low vs. high), no significant differences were found. This indicates that heterogeneity in the results is not explained by these factors, but may stem from other unmeasured variables [48]. The sensitivity analysis revealed that the exclusion of studies, such as Kumar et al. [22] and Lokireddy et al. [31], significantly impacted the pooled OR, highlighting that some studies exerted more influence on the overall effect than others. These findings emphasize the importance of considering individual study characteristics when interpreting meta-analysis results [49].

Overall, these findings emphasize caution in generalizing the role of ABO and Rh blood groups in CHIKV infection risk. Based on current evidence, ABO and Rh blood groups do not appear to be reliable markers for risk stratification in CHIKV infection. Understanding host genetic factors remains crucial for the development of predictive models of CHIKV epidemiology and clinical outcome [50]. Future research should integrate molecular virology, immunogenetics, and large-scale, population-specific studies to validate mechanistic hypotheses and uncover possible interactions between blood group antigens and specific CHIKV strains. Additionally, viral binding assays or studies exploring glycan-mediated entry may shed light on the functional role of blood group antigens in CHIKV pathogenesis.

This study has several limitations. First, the high heterogeneity observed across studies limits the precision of pooled effect estimates, weakening the robustness of the findings. Second, the use of the STROBE checklist as a scale for study quality is limited, as not all of its items directly relate to internal validity, which could affect the strength of the evidence. Additionally, the detected publication bias may have influenced the results. Finally, the exclusion of a single outlier study led to non-significant results, suggesting that the overall findings could have been influenced by the inclusion of certain studies.

## 5. Conclusions

Current evidence indicates a significant association between blood group O and increased susceptibility to Chikungunya virus infection. In contrast, no consistent associations were observed for other ABO or Rh(D) blood groups. However, these findings should be interpreted with caution due to substantial heterogeneity and methodological limitations in the included studies. Further large-scale, population-specific studies with standardized diagnostic criteria and robust blood group determination are needed to clarify these associations and explore underlying biological mechanisms.

## Data Availability

The supporting data for the findings of this study can be obtained by contacting the corresponding author upon request.

## Supplementary Materials

The following supporting information can be downloaded at: https://www.mdpi.com/article/10.3390/medicina61081316/s1, Table S1: Search strategy; Table S2: Quality assessment; Table S3: Study characteristics; Figure S1: Subgroup analysis of ABO blood group, based on study design; Figure S2: Subgroup analysis of ABO blood group, based on study quality; Figure S3: Subgroup analysis of Rh blood group; Figure S4: Funnel plot analysis.

## Abbreviations

The following abbreviations are used in this manuscript:

ACE2	Angiotensin-converting enzyme 2
CHIKV	Chikungunya virus
CI	Confidence interval
*I*^2^	Inconsistency index
MeSH	Medical Subject Headings
NSAIDs	Nonsteroidal anti-inflammatory drugs
OR	Odds ratio
PEO	Patient, Exposure, and Outcome
PRISMA	Preferred Reporting Items for Systematic Reviews and Meta-Analyses
Rh	Rhesus
SARS-CoV-1	Severe acute respiratory syndrome coronavirus 1
SARS-CoV-2	Severe acute respiratory syndrome coronavirus 2
STROBE	Strengthening the Reporting of Observational Studies in Epidemiology
