# Supplementary material for: Association Between ABO or Rh Blood Groups and Chikungunya Virus Infection: A Systematic Review and Meta-Analysis
Source: Medicina (Kaunas). 2025 Jul 22;61(8):1316. doi: 10.3390/medicina61081316 (PMC12387744; doi:10.3390/medicina61081316)
Supplement: Supplementary file 1 [file medicina-61-01316-s001.zip › Supplementary figures.pdf]

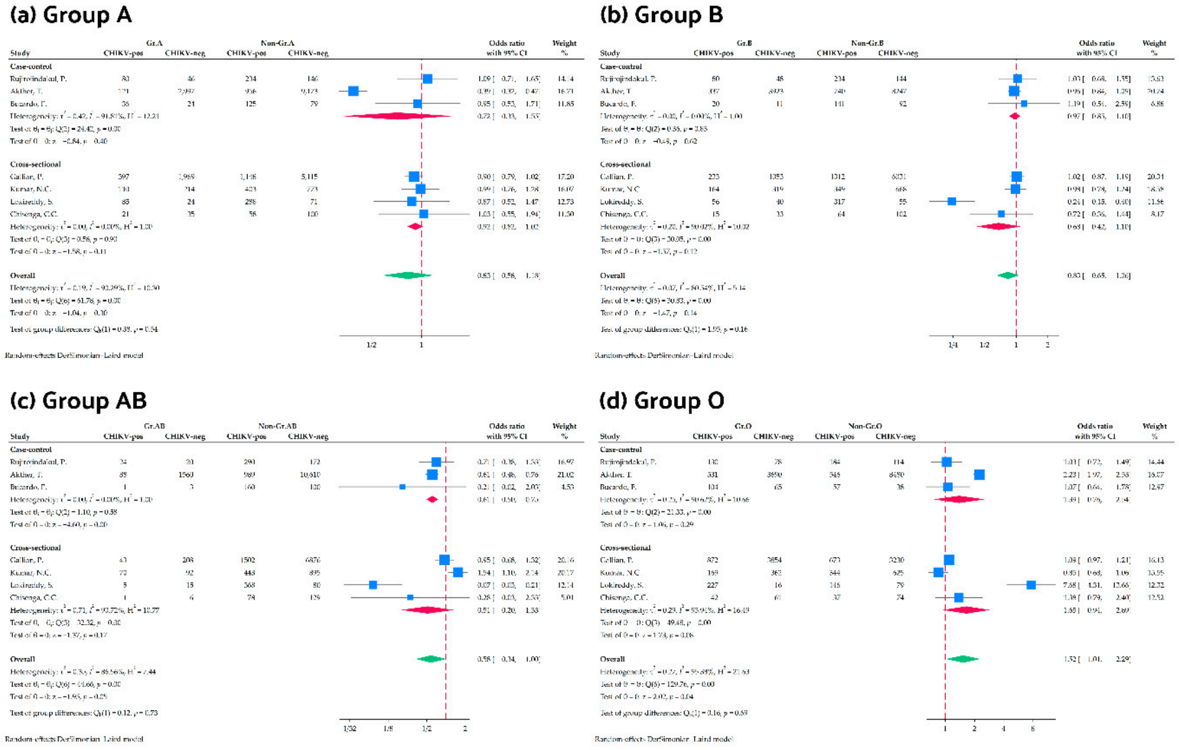

Figure S1. Subgroup analysis of association between ABO blood groups and CHIKV infection, based on study design (case-control vs. cross-sectional) for blood groups (a) A, (b) B, (c) AB, and (d) O, respectively.

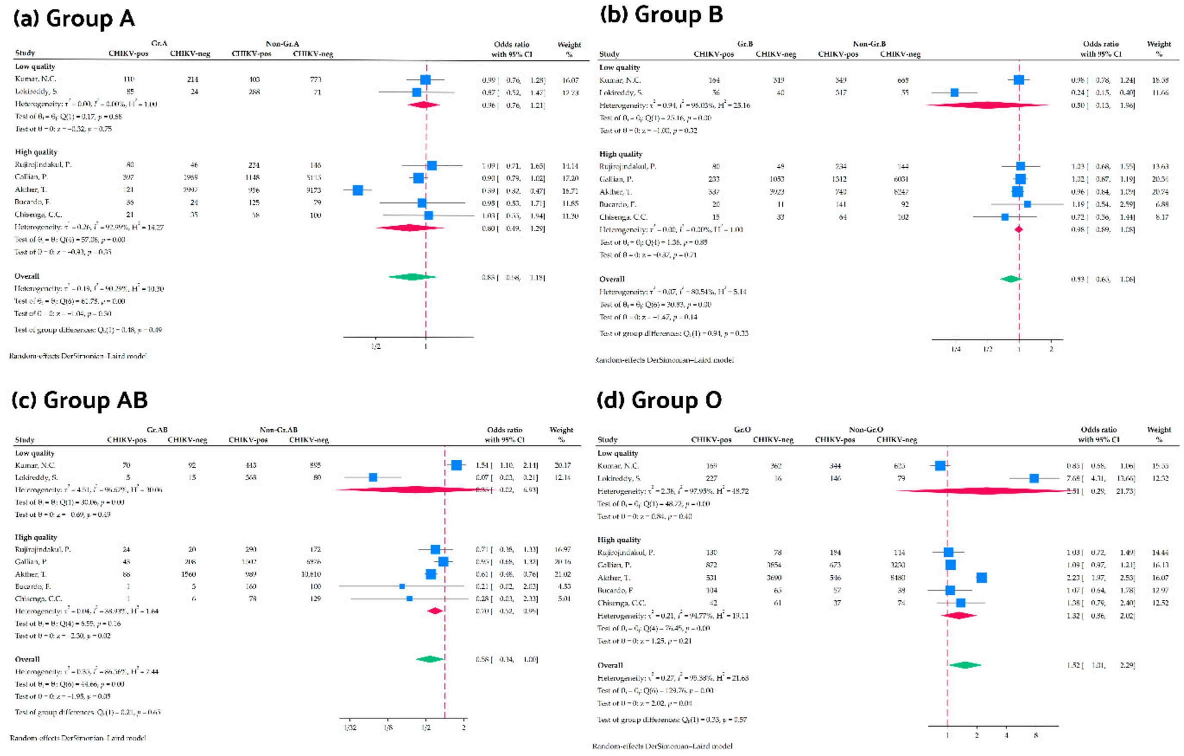

Figure S2. Subgroup analysis of association between ABO blood groups and CHIKV infection, based on study quality (low vs. High) for blood groups (a) A, (b) B, (c) AB, and (d) O, respectively.

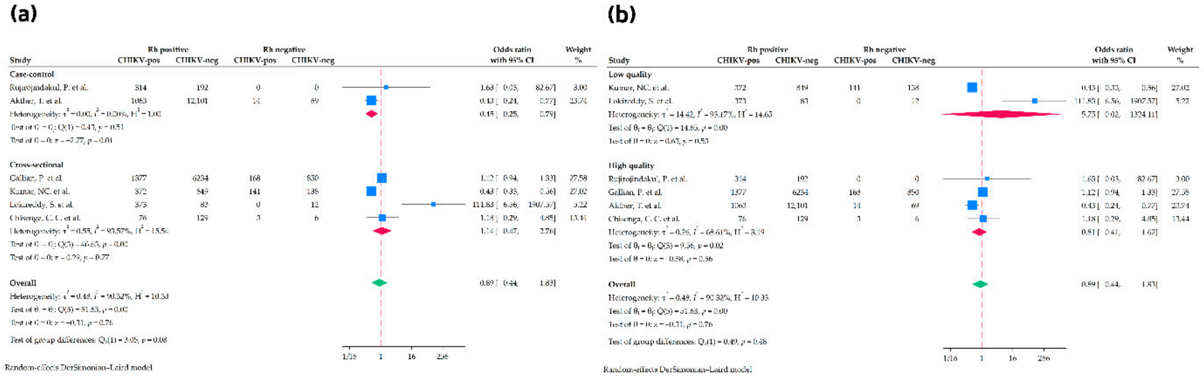

Figure S3. Subgroup analysis of association between Rh blood groups and CHIKV infection, based on (a) study design (case-control vs. cross-sectional) and (b) study quality (low vs. high).

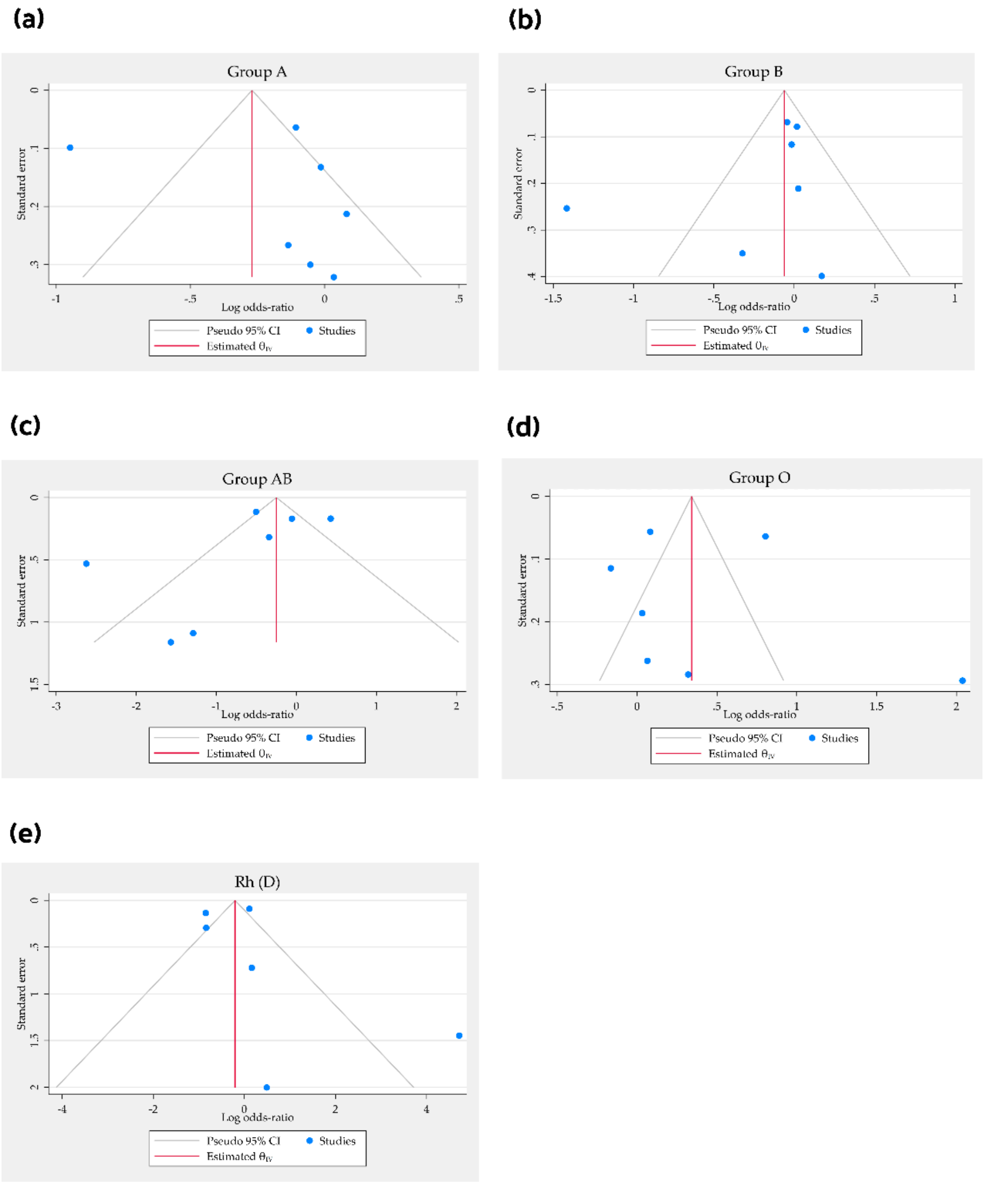

Figure S4. Funnel plots illustrate publication bias for blood groups (a) A, (b) B, (c) AB, (d) O, and (e) Rh(D).
