# Supplementary material for: Association Between ABO or Rh Blood Groups and Chikungunya Virus Infection: A Systematic Review and Meta-Analysis
Source: Medicina (Kaunas). 2025 Jul 22;61(8):1316. doi: 10.3390/medicina61081316 (PMC12387744; doi:10.3390/medicina61081316)
Supplement: Supplementary file 1 [file medicina-61-01316-s001.zip › Table S1 Search strategy.pdf]

Table S.1 Search Strategy

1. PubMed (January 23, 2025)

Keyword	Search strategy	Search result
Chikungunya	"chikungunya fever"[MeSH Terms] OR "Chikungunya"[Text Word] OR "chikv"[Text Word] OR "chik virus"[Text Word]	8,360
Blood group	"blood group antigens"[MeSH Terms] OR "abo blood group system"[MeSH Terms] OR "abo blood group"[Text Word] OR "rh hr blood group system"[MeSH Terms] OR "rh blood group"[Text Word] OR "rh antigen*"[Text Word] OR "blood group*"[Text Word]	60,691
Combined	("chikungunya fever"[MeSH Terms] OR "Chikungunya"[Text Word] OR "chikv"[Text Word] OR "chik virus"[Text Word]) AND ("blood group antigens"[MeSH Terms] OR "abo blood group system"[MeSH Terms] OR "abo blood group"[Text Word] OR "rh hr blood group system"[MeSH Terms] OR "rh blood group"[Text Word] OR "rh antigen*"[Text Word] OR "blood group*"[Text Word])	9

2. Scopus (January 23, 2025)

Keyword	Search strategy	Search result
Chikungunya	(TITLE-ABS-KEY ("Chikungunya") OR TITLE-ABS-KEY (chikv) OR TITLE-ABS-KEY ("chik virus"))	12,048

Keyword	Search strategy	Search result
Blood group	(TITLE-ABS-KEY ("Blood group*") OR TITLE-ABS-KEY ("ABO blood group*") OR TITLE-ABS-KEY ("Rh blood group") OR TITLE-ABS-KEY ("Rh antigen*") OR TITLE-ABS-KEY ("A antigen") OR TITLE-ABS-KEY ("B antigen") OR TITLE-ABS-KEY ("D antigen"))	108,648
Combined	((TITLE-ABS-KEY ("Chikungunya") OR TITLE-ABS-KEY (chikv) OR TITLE-ABS-KEY ("chik virus"))) AND ((TITLE-ABS-KEY ("Blood group*") OR TITLE-ABS-KEY ("ABO blood group*") OR TITLE-ABS-KEY ("Rh blood group") OR TITLE-ABS-KEY ("Rh antigen*") OR TITLE-ABS-KEY ("A antigen") OR TITLE-ABS-KEY ("B antigen") OR TITLE-ABS-KEY ("D antigen")))	28

3. MEDLINE (January 23, 2025)

Keyword	Search strategy	Search result
Chikungunya	AB (Chikungunya OR chikv OR chik virus)	7,123
Blood group	AB Blood group OR ABO antigen OR Rh blood group OR Rh antigen OR Rh antigen OR A antigen OR B antigen)	1,058,383
Combined	AB (Blood group OR ABO antigen OR Rh blood group OR Rh antigen OR Rh antigen OR A antigen	366

	OR B antigen) AND AB (Chikungunya OR chikv OR chik virus)	

4. EMBASE (January 23, 2025)

**Keyword**	**Search strategy**	**Search result**
Chikungunya	'chikungunya'/exp OR chikungunya OR chikv OR 'chik virus'	12,437
Blood group	'blood group'/exp OR 'blood group' OR 'rbc antigen' OR 'abo antigen' OR 'blood group abo system'/exp OR 'blood group abo system' OR 'blood group rhesus system'/exp OR 'blood group rhesus system' OR 'rhesus antigen'/exp OR 'rhesus antigen'	80,777
Combined	('chikungunya'/exp OR chikungunya OR chikv OR 'chik virus') AND ('blood group'/exp OR 'blood group' OR 'rbc antigen' OR 'abo antigen' OR 'blood group abo system'/exp OR 'blood group abo system' OR 'blood group rhesus system'/exp OR 'blood group rhesus system' OR 'rhesus antigen'/exp OR 'rhesus antigen')	19

5. Ovid (January 23, 2025)

**Keyword**	**Search strategy**	**Search result**
Chikungunya	Chikungunya.mp. [mp=tx, bt, ti, ot, ab, ct, sh, kw, fx, hw, nm, kf, ox, px, rx, an, ui, ds, on, sy, ux, mx] OR chikv.mp. [mp=tx, bt, ti, ot, ab, ct, sh, kw, fx, hw, nm, kf, ox, px, rx, an, ui, ds, on, sy, ux, mx] OR 'chik	14,739

Keyword	Search strategy	Search result
	virus'.mp. [mp=tx, bt, ti, ot, ab, ct, sh, kw, fx, hw, nm, kf, ox, px, rx, an, ui, ds, on, sy, ux, mx]	
Blood group	'blood group*'.mp. [mp=tx, bt, ti, ot, ab, ct, sh, kw, fx, hw, nm, kf, ox, px, rx, an, ui, ds, on, sy, ux, mx] OR 'rbc antigen*'.mp. [mp=tx, bt, ti, ot, ab, ct, sh, kw, fx, hw, nm, kf, ox, px, rx, an, ui, ds, on, sy, ux, mx] OR 'abo antigen*'.mp. [mp=tx, bt, ti, ot, ab, ct, sh, kw, fx, hw, nm, kf, ox, px, rx, an, ui, ds, on, sy, ux, mx] OR 'Rh antigen*'.mp. [mp=tx, bt, ti, ot, ab, ct, sh, kw, fx, hw, nm, kf, ox, px, rx, an, ui, ds, on, sy, ux, mx] OR 'Rhesus antigen*'.mp. [mp=tx, bt, ti, ot, ab, ct, sh, kw, fx, hw, nm, kf, ox, px, rx, an, ui, ds, on, sy, ux, mx]	94,123
Combined	(Chikungunya.mp. [mp=tx, bt, ti, ot, ab, ct, sh, kw, fx, hw, nm, kf, ox, px, rx, an, ui, ds, on, sy, ux, mx] OR chikv.mp. [mp=tx, bt, ti, ot, ab, ct, sh, kw, fx, hw, nm, kf, ox, px, rx, an, ui, ds, on, sy, ux, mx] OR 'chik virus'.mp. [mp=tx, bt, ti, ot, ab, ct, sh, kw, fx, hw, nm, kf, ox, px, rx, an, ui, ds, on, sy, ux, mx]) AND ('blood group*'.mp. [mp=tx, bt, ti, ot, ab, ct, sh, kw, fx, hw, nm, kf, ox, px, rx, an, ui, ds, on, sy, ux, mx] OR 'rbc antigen*'.mp. [mp=tx, bt, ti, ot, ab, ct, sh, kw, fx, hw, nm, kf, ox, px, rx, an, ui, ds, on, sy, ux, mx] OR 'abo antigen*'.mp. [mp=tx, bt, ti, ot, ab, ct, sh, kw, fx, hw, nm, kf, ox, px, rx, an, ui, ds, on, sy, ux, mx] OR 'Rh antigen*'.mp. [mp=tx, bt, ti, ot, ab, ct, sh, kw, fx, hw, nm, kf, ox, px, rx, an, ui, ds, on, sy, ux, mx] OR 'Rhesus antigen*'.mp. [mp=tx, bt, ti, ot, ab, ct, sh, kw, fx, hw, nm, kf, ox, px, rx, an, ui, ds, on, sy, ux, mx])	74

6. ProQuest (July 8, 2025)

Keyword	Search strategy	Search result
Chikungunya	chikungunya OR CHIKV	3,829
Blood group	"Blood group*" OR ABO OR Rh AND Rhesus	79,943
Combined	[chikungunya OR CHIKV] AND ["Blood group*" OR ABO OR Rh AND Rhesus]	416

7. Google Scholar (July 8, 2025)

Keyword	Search strategy	Search result
Combined	"Blood group" AND Chikungunya	100
