# Supplementary figures and images for: Association Between ABO or Rh Blood Groups and Chikungunya Virus Infection: A Systematic Review and Meta-Analysis
Source: Medicina (Kaunas). 2025 Jul 22;61(8):1316. doi: 10.3390/medicina61081316 (PMC12387744; doi:10.3390/medicina61081316)

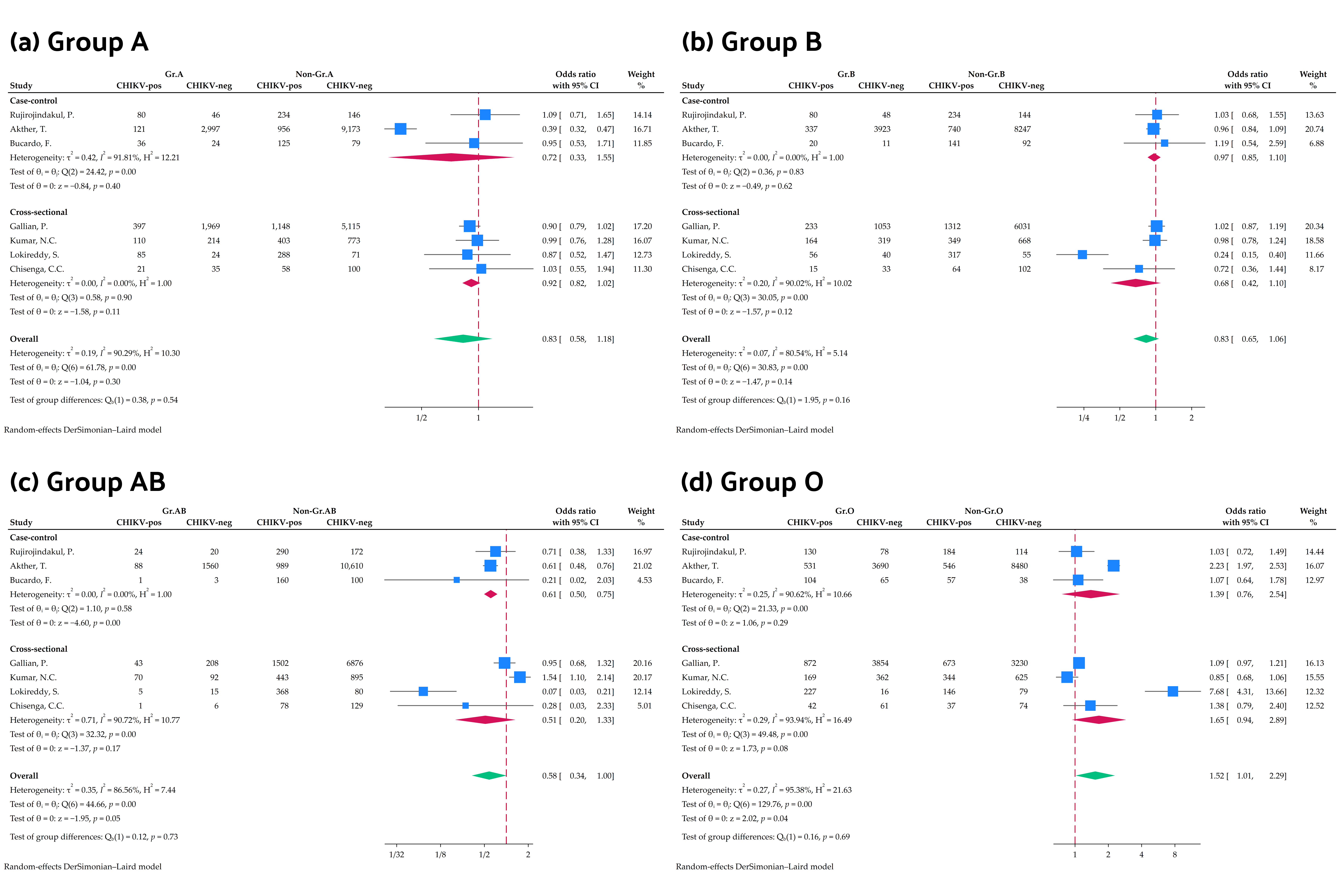

Supplement: Supplementary file 1 [file medicina-61-01316-s001.zip › Figure S1.png]

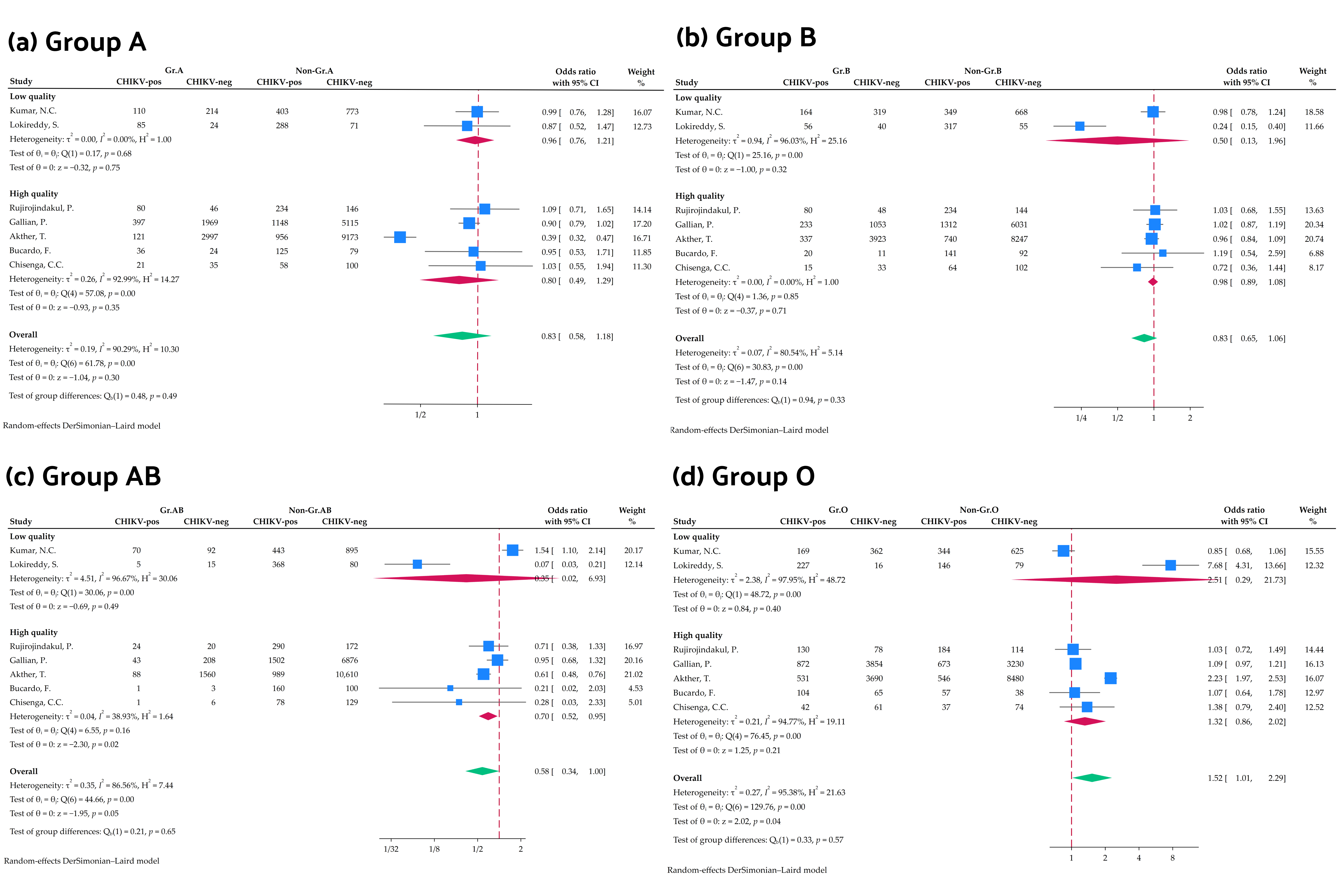

Supplement: Supplementary file 1 [file medicina-61-01316-s001.zip › Figure S2.png]

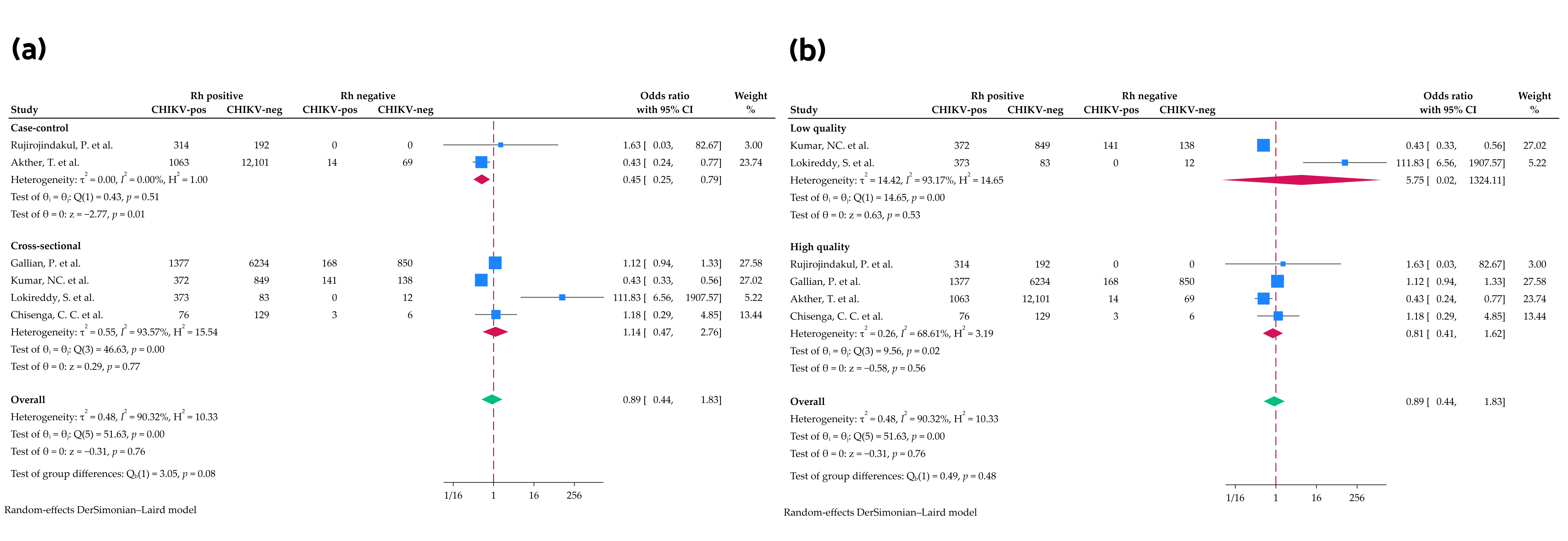

Supplement: Supplementary file 1 [file medicina-61-01316-s001.zip › Figure S3.png]

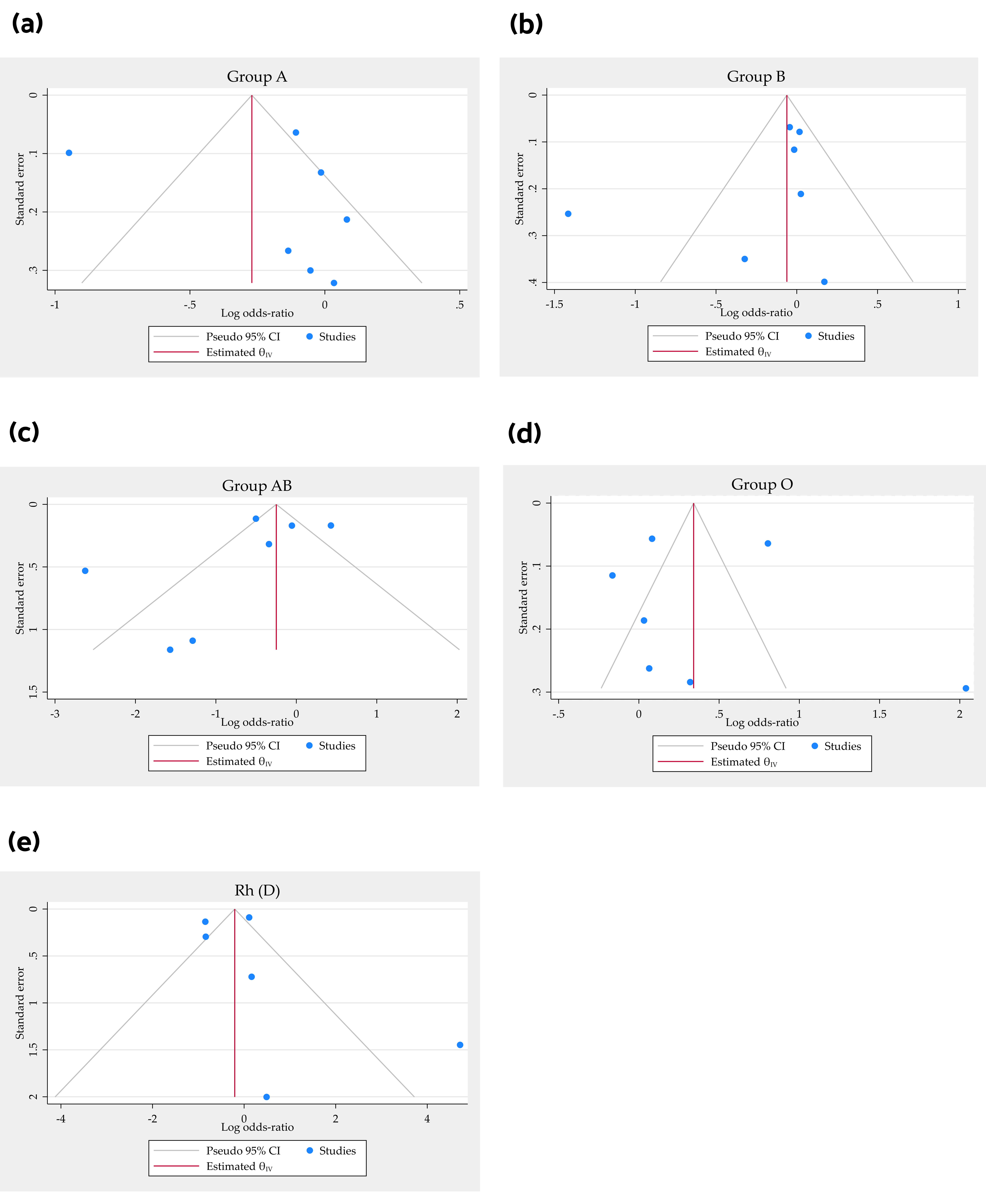

Supplement: Supplementary file 1 [file medicina-61-01316-s001.zip › Figure S4.png]
